# Molecular characterization of occult hepatitis B virus infection in patients with end-stage liver disease in Colombia

**DOI:** 10.1371/journal.pone.0180447

**Published:** 2017-07-07

**Authors:** Julio Cesar Rendon, Fabian Cortes-Mancera, Juan Carlos Restrepo-Gutierrez, Sergio Hoyos, Maria-Cristina Navas

**Affiliations:** 1 Grupo de Gastrohepatologia, Facultad de Medicina, Universidad de Antioquia, UdeA, Medellin, Colombia; 2 Grupo de Investigación e Innovacion Biomédica GI^2^B, Facultad de Ciencias Exactas y Aplicadas, Instituto Tecnologico Metropolitano (ITM), Medellin, Colombia; 3 Unidad de Hepatologia y Trasplante Hepatico, Hospital Pablo Tobon Uribe, Medellin, Colombia; Centre de Recherche en Cancerologie de Lyon, FRANCE

## Abstract

**Background:**

Hepatitis B virus (HBV) occult infection (OBI) is a risk factor to be taken into account in transfusion, hemodialysis and organ transplantation. The aim of this study was to identify and characterize at the molecular level OBI cases in patients with end-stage liver disease.

**Methods:**

Sixty-six liver samples were obtained from patients with diagnosis of end-stage liver disease submitted to liver transplantation in Medellin (North West, Colombia). Samples obtained from patients who were negative for the surface antigen of HBV (n = 50) were tested for viral DNA detection by nested PCR for ORFs S, C, and X and confirmed by Southern-Blot. OBI cases were analyzed by sequencing the viral genome to determine the genotype and mutations; additionally, viral genome integration events were examined by the Alu-PCR technique.

**Results:**

In five cases out of 50 patients (10%) the criteria for OBI was confirmed. HBV genotype F (subgenotypes F1 and F3), genotype A and genotype D were characterized in liver samples. Three integration events in chromosomes 5q14.1, 16p13 and 20q12 affecting Receptor-type tyrosine-protein phosphatase T, Ras Protein Specific Guanine Nucleotide Releasing Factor 2, and the zinc finger 263 genes were identified in two OBI cases. Sequence analysis of the viral genome of the 5 OBI cases showed several punctual missense and nonsense mutations affecting ORFs S, P, Core and X.

**Conclusions:**

This is the first characterization of OBI in patients with end-stage liver disease in Colombia. The OBI cases were identified in patients with HCV infection or cryptogenic cirrhosis. The integration events (5q14.1, 16p13 and 20q12) described in this study have not been previously reported. Further studies are required to validate the role of mutations and integration events in OBI pathogenesis.

## Introduction

Hepatitis B virus (HBV) is responsible for significant morbidity and mortality worldwide. It is estimated that one-third of the world population has been infected by HBV and at least 240 million individuals are chronic carriers of the virus. Additionally, more than 780.000 people die each year due to liver diseases associated with HBV infection including cirrhosis and hepatocellular carcinoma (HCC) [1, 2].

HBV infection is diagnosed by detecting the HBV surface antigen (HBsAg) in serum samples using immunoassays; although other serological markers are necessary to identify the type and/or stage of HBV infection including the E antigen (HBeAg), anti-Core protein (anti-HBc), anti-HBsAg (anti-HBs) and anti-HBeAg (anti-HBe) antibodies and viral load [3, 4].

In 1979 evidence of a particular type of HBV infection was described [5], that was subsequently recognized as occult HBV infection (OBI) [6]. OBI is a persistent infection characterized by the absence or undetected HBsAg in the serum, viral load <1000 copies/mL, and with or without detection of other serological markers [7]. Patients with OBI could develop HBV reactivation, cirrhosis and HCC [8, 9].

OBI is a multifactorial pathology in which are involved mutations affecting viral proteins and promoters, Hepatitis C Virus (HCV) and/or Human Immunodeficiency Virus (HIV) coinfection, host immune response, and cellular defense mechanisms such as epigenetic silencing of the viral genome [10–12].

OBI has been reported in populations with risk factors including hemodialysis [13, 14], HIV infection [15–18], HCV infection [19–21], HCC [22–24], cryptogenic liver disease [25, 26], and end-liver disease [27, 28]. A large variation in OBI frequency has been reported in the different studies depending on the HBV prevalence in the general population, the risk factor and the criteria for OBI (13–28).

Recently, 8 studies of OBI were carried out in Colombia and have shown a prevalence of OBI from 1% to 8.3% in HIV patients [29–31], 0% to 8.75% in blood donors [32–34], 0% in undergraduate student populations [35] and 13.3% in patients with diagnosis of cirrhosis and/or HCC [36]. However, there are some limitations in these studies, such as size sample, HBV genome detection techniques and OBI criteria. Thus, additional studies are necessary to establish the prevalence of OBI in the Colombian population.

OBI in recipients of liver transplants is a risk factor to be taken into account, considering *de novo* infections that occur in 25–95% of transplants from HBsAg negative, anti-HBc positive donors [37–39]. According to the Department of Health and Human Resources, in the USA around 79 persons receive organ transplant per day, liver transplantation being approximately 21% of all procedures [40]; meanwhile in Colombia in 2014, a total of 1046 transplant procedures were made, of which 211 (20%) were liver transplants [41].

Evidence has shown that in solid organ transplantations such as kidney [42], bone marrow [43], and heart [44], HBV transmission is usually lower than 5%; while for recipients of liver transplants, a variable rate of *de novo* HBV infection occurs between 17% and 95% depending on the HBV serological markers of the donor [27, 37, 38, 45, 46]. However, the infection status of the recipient is also highly relevant, considering that among 4.4% and 17.6% of OBI has been reported in liver transplant recipients [28, 47–49].

The aim of this study was to determine the frequency of OBI in a cohort of patients with end-stage liver disease, submitted to liver transplantation, attending a hospital in Medellin, and to characterize mutations in the viral genome and viral integration events that could be related with OBI.

## Material and methods

### Study population

#### Specimen collection

This study included 66 liver tissue samples, obtained between August 2002 and February 2008, from patients with diagnosis of end-stage liver disease submitted to liver transplantation at the Pablo Tobon Uribe Hospital, one of the most important centers of liver transplantation in Colombia. The samples were collected in dry sterile 50 mL tubes at the time of surgery, conserved at 4°C and transported at 4°C to the laboratory within 12 hours to 24 hours after collection, where samples were aliquoted in independent containers and conserved at -70°C. In few cases, the liver sample was sent to the laboratory 24–48 hours after surgery.

Cirrhosis and HCC were diagnosed based on the criteria of the European Association for the Study of the Liver (EASL) and the American Association for the Study of Liver Diseases (AASLD) [50, 51].

The OBI cases were defined, based on criteria described by Brechot et al., as HBsAg negative cases in which amplification of at least two different HBV genome regions was confirmed [52].

### Hepatic and viral markers

Quantification of serological markers was performed using the platform ARCHITECT ci8200 (Abbott, USA). Serological markers of liver function such as Alanine Aminotransferase (ALT), and Aspartate Aminotransferase (AST) were quantified using the Activated alanine or aspartate aminotransferase assay (Abbott, USA) while serological HBV infection markers were quantified using the chemiluminescent microparticle immunoassay (Abbott, USA).

### HBV genome detection

From cases negative for HBsAg (n = 50), total DNA was extracted from liver tissue using the QIAamp DNA Mini Kit (QIAGEN, Germany) according to the manufacturer’s protocol. DNA was quantified using the NanoDrop ND 1000 Spectrophotometer (Thermo Fisher Scientific, USA), and quality tested by amplification of GAPDH gene [53]. DNA (60–80 ηg) was used to amplify three regions of the HBV genome (X, S and Core-X regions) by nested or hemi-nested PCR strategies as described previously (S1 Table).

Amplification of the ORF S was performed using 1 unit of Biolase DNA polymerase (Bioline, USA), 5 mM of MgCl_2_, and 10 pmol of primers YS1 and YS2 [54]. The second round was performed using 1.25 U of Biolase DNA polymerase (Bioline, USA), 1 mM of MgCl_2_ and 10 pmol of primers S3s and S3as [55]. Cycling of the first round included an initial denaturation step at 95°C for 3 min and 35 cycles at 94°C for 45 s, 53°C for 60 s and 72°C for 90 s; the second round included an initial denaturation step at 95°C for 3 min and 35 cycles at 94°C for 45 s, 50°C for 60 s, and 72°C for 60 s and one cycle at 72°C for 2 min.

PCR reaction of ORF X was performed using 1.5 unit of Biolase DNA polymerase, 1.5 mM of MgCl_2_ and 10 pmol of primers X1F and X3R. The second round was carried out in the same conditions using primers X1F and X1R [56, 57]. Cycling of both rounds included an initial denaturation step at 95°C for 3 min and 30 cycles at 94°C for 45 s, 53°C for 30 s, and 72°C for 40 s, and one cycle at 72°C for 2 min.

The X-Core region was amplified using a hemi-nested PCR. In this strategy both rounds were performed using 1 unit of Biolase DNA polymerase, 1 mM of MgCl_2_ and 7.5 pmol of primers 1101p and 2440n [58]. The second round was carried out in the same conditions using primers P2 and 1101p. The cycling conditions for the first round included an initial denaturation step at 95°C for 3 min and 35 cycles of 94°C for 45 s, 55°C for 45 s, and 72°C for 90 s; the second round included an initial denaturation at 95°C for 3 min and 35 cycles at 94°C for 45 s, 55°C for 45 s and 72°C for 60 s and one cycle at 72°C for 2 min.

### Southern blot analysis

A full length HBV DNA (3,2 kb) was amplified from the sample TH24 (previously designated FJ589065.1) [59] using the method described by Gunther et al., [60]. The amplification products were purified using the GeneJet Gel Extraction kit (Thermo Fisher Scientific, USA), and cloned into the vector pJET1.2 (Thermo Fisher Scientific, USA) to create the construct pJET-TH24_1,5.

DNA (40–60 μg) obtained from liver samples and 1 μg of the pJET-TH24_1,5 construct were pretreated with *EcoR*I restriction enzyme (Thermo Fisher Scientific, USA), and separated by electrophoresis on a 1% agarose gel. The gel was blotted onto a nylon membrane (Roche Diagnostics GmbH, Germany) and hybridized with a digoxigenin (DIG)-labeled probe specific for the HBV S region (nucleotides 422–759). The probe was generated using the primers S3s-S3as (S1 Table) and the plasmid pJET-TH24_1,5 with a commercial kit (PCR DIG Probe Synthesis Kit; Roche Diagnostics GmbH, Germany). The hybridization signals were visualized using an enzyme-linked immunoassay (DIG Luminescent Detection Kit; Roche Diagnostics GmbH, Germany) following the protocol recommended by the supplier.

### Complete genome sequence

Four overlapping fragments of the HBV genome were amplified using 80 ng of total DNA extracted from the OBI samples, using the HotStart HiFidelity Polymerase kit (Qiagen, Germany) and the following primer sets 58p-1450n, 58n-2440p, P2-1101P and P1-2440n (S1 Table and S1 Fig) [60, 61]. The PCR products were purified using the GeneJet Gel Extraction kit (Thermo Fisher Scientific, USA) and cloned into the cloning vector pJET1.2 (Thermo Fisher Scientific, USA) according to the manufacturer’s indications. Cloned PCR products were sequenced (Macrogen, Inc., Seoul, Korea) and then analyzed using the ContigExpress application of Vector NTI 11.0 (Invitrogen, USA).

The nucleotide sequence data are available in the GeneBank databases (TH3: KX757662, KX757663, KX757664; TH6: KX75766; TH28: KX757666, KX757667; TH75: KX757668; and TH78: KX757670, KX757671, KX757672).

### Phylogenetic analysis

HBV genome sequences from the OBI cases were analyzed by phylogenetic reconstruction using 118 HBV sequences including 32 partial and 86 HBV full-length genome sequences (S2 Table) available in GenBank.

The sequences were aligned and edited using BioEdit 7.2 software [62] (available at http://www.mbio.ncsu.edu/bioedit/bioedit.html). The phylogenetic analyses were conducted using the MEGA software version 6.06. The phylogenetic tree was constructed using Neighbor-joining method and GTR model; the bootstrap consensus tree was inferred from 1000 replicates [63]. Pairwise distance test was performed to ensure no contamination between samples.

### Pairwise and mutation analysis

Sequences of OBI cases were grouped according to the genotype identified by the phylogenetic analysis, and aligned with 3 HBV full-length genome sequences of genotypes A, D, and F, chosen randomly from the database of reference sequences used for the phylogenetic analysis. None of these sequences were related to OBI (Genotype A: AJ012207, AJ309369 and AF090838; genotype D: AF280817, AB033558 and AB078033; Genotype F: FJ58065, DQ899145 and AY179734, S3 Table).

Based on the DNA sequence, each ORF sequence was aligned, and the deduced amino acid sequence was analyzed. OBI mutations were considered based on previously reported mutations and new mutations identified in OBI sequences compared to the reference sequences of each genotype.

### Viral genome integration

HBV DNA integration into the host genome was examined using the Alu-PCR methodology described by Minami et al. and Murakami et al. with some modifications [64, 65]. Briefly, 100 ηg of total DNA extracted from liver samples were amplified in a final volume of 50 μL containing, 1 unit of Biolase DNA polymerase, 1 mM of MgCl_2_, 50 pmol of Alu primer and 10–100 pmol of primer HB1 (S1 Table). The cycling conditions included an initial denaturation step at 94°C for 3 min and 10 cycles of 94°C for 30 s, 59°C for 30 s, and 70°C for 3 min.

The PCR products were then incubated with 1 U of uracil-DNA glycosylase (Life Technologies, USA) for 30 min at 37°C followed by 10 min at 94°C. Subsequently, 1 unit of Biolase DNA polymerase and 10 mmol of HB2 and Tag5 primers (S1 Table) were added and a Touch Down PCR was carried out. The cycling condition for the first step was 94°C during 3 min, followed by 20 cycles at 94°C for 30 s, 65°C for 30 s decreasing 0.5°C/cycle, 70°C for 3 min, and finally 20 cycles at 94°C for 30 s, 55°C for 30 s, and 70°C for 3 min, followed by one final step at 72°C for 8 min. Finally, 5 μl of the amplified product was used for a second round of amplification using the HB2 and Tag5 primers under the follow conditions: 20 cycles at 94°C for 30 s, 55°C for 30 s, and 70°C for 3 min and one final step at 72°C for 8 min.

The PCR products were analyzed on 1% agarose gels stained with ethidium bromide; the bands were purified using QIAquick gel extraction kit (QIAGEN, Germany) and then sequenced by the Sanger sequencing method (Macrogen, Inc., Seoul, Korea).

The sequences were analyzed using BioEdit software and ContigExpress module from Vector NTI 4.1 (Invitrogen, USA); identification of integration events was made using BLASTn search system.

### Statistical analysis

Normality was assessed by the Shapiro-Wiks test and the *χ*^2^ test, Student´s *t*-test and the Mann-Whitney *U* test were used to identify correlations between parameters. Calculations were performed with the SPSS software package 23. A *p <*0.05 was considered significant.

### Ethics considerations

The study was approved by the Ethics Committees of Universidad de Antioquia (N 007, Facultad de Medicina, UdeA) and the Hospital Pablo Tobon Uribe (No. 13–2012, HPTU). None of the transplant donors were from vulnerable population and all donors provided written informed consent that was freely given.

## Results

### Study population

The study was carried on liver tissue samples obtained from 66 patients, 39 (39/66, 59.1%) male and 27 (40.9%) female. Forty nine patients (74.2%) had been diagnosed of cirrhosis without HCC; among them 10 (20.4%) had cryptogenic cirrhosis; 9 (18.3%) had cirrhosis associated with HCV infection, 8 (16.3%) cirrhosis associated with cholangiopathies, 7 (14.2%) cirrhosis associated with autoimmune hepatitis, 6 (12.2%) cirrhosis associated with HBV infection, 4 (8.16%) had alcoholic cirrhosis, 2 (4.1%) alcohol and HBV or HCV infection, 1 (2%) HBV/HCV coinfection and 2 (4.1%) related to nonalcoholic steatohepatitis (NASH) or Wilson disease.

Moreover, the 17 (25.8%) cases of cirrhosis and HCC were associated with HBV infection (5/17, 29.4%), HCV infection (5/17, 29.4%), HBV/HCV coinfection (4/17, 23.5%) and autoimmune hepatitis (2/17, 11.8%); In addition, 1 cryptogenic case was identified (1/17, 5.8%) (Table 1). Among all cases, only samples related with autoimmune hepatitis presented a significant correlation with the gender (p<0.003).

**Table 1:** Demographical and clinical characteristics of patients with end-stage liver disease submitted to liver transplantation.

Etiology	Cirrhosis Male/Female Mean Age (Range)	Cirrhosis and HCC Male/Female Mean Age (Range)	Total Male/Female Mean Age (Range)
Cryptogenic	n = 10 7/3 52.1 (29–71)	n = 1 0/1 65	n = 11 7/4 54.5 (29–71)
HBV infection	n = 6 6/0 54.5 (46–63)	n = 5 3/2 56.6 (45–67)	n = 11 9/2 53.67 (45–67)
HCV infection	n = 9 7/2 53.8 (34–68)	n = 5 3/2 54 (44–63)	n = 14 10/4 55.4 (34–68)
HBV- HCV coinfection	n = 1 0/1 58	n = 4 2/2 62.5 (54–68)	n = 5 2/3 61.6 (54–68)
Alcohol	n = 4 4/0 55 (51–64)	n = 0	n = 4 4/0 55 (51–64)
Alcohol + HBV or HCV	n = 2 1/1 46.5 (34–69)	n = 0	n = 2 1/1 44.2 (34–69)
Autoimmune hepatitis ^a^	n = 7 0/7 44.7 (25–66)	n = 2 1/1 42.5 (32–53)	n = 9 1/8 41.5 (25–66)
Cholangiopathies	n = 8 4/4 45.6 (34–57)	n = 0	n = 8 4/4 45.6 (34–57)
Others ^b^	n = 2 1/1 38 (17–59)	n = 0	n = 2 1/1 38 (17–59)
Total	n = 49 30/19 50.2 (17–71)	n = 17 9/8 56.1 (32–68)	n = 66 39/27 51.7 (17–71)

^a^ Including three patient with primary biliary cirrhosis and autoimmune hepatitis

^b^ Once case of Wilson disease and one of NASH

**Fig 1:**
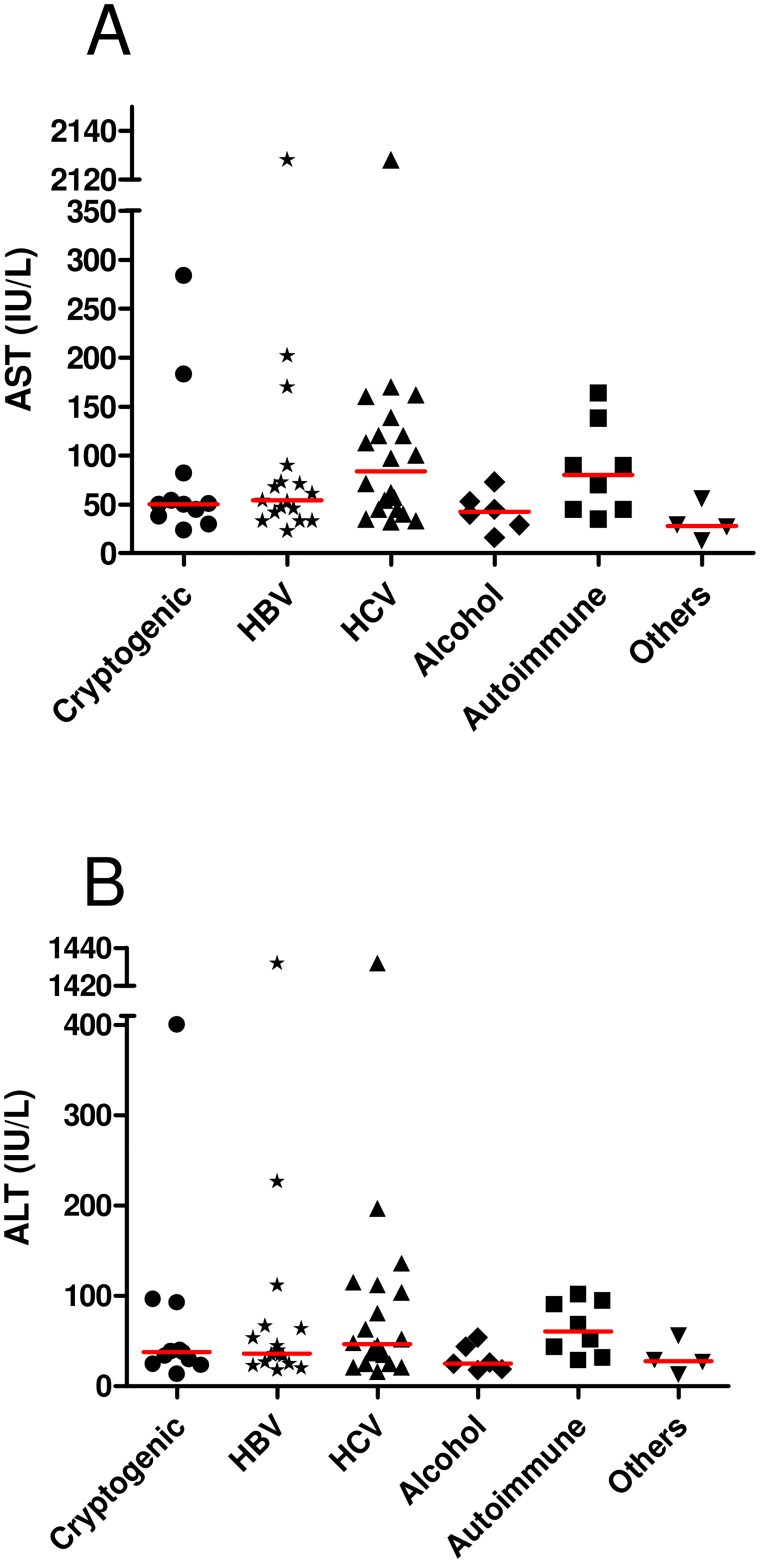
AST and ALT serum levels of the study pupulation. AST (A) and ALT (B) serum levels before transplant of all patients. Red lines indicates the median value.

No differences in ALT and AST serum levels were found among the patients with different liver damage etiologies. Meanwhile, AST was significantly higher in patients with cholangiopathies, 90.5 U/L compared with other etiologies 57.5 U/L (p = 0.029). Additionally, AST and ALT serum levels were lower in alcohol consuming patients (42.5 U/L and 25.5 U/L) compared with patients without alcohol consumption (61.5U/L and 44 U/L) (p = 0.039 and p = 0.031, respectively) (Table 2, Fig 1).

**Table 2:** AST and ALT serum levels in patients with end-stage liver disease and submitted to liver transplantation.

Variable	Category (n)	AST (U/L)		ALT (U/L)	
		Median (Range)	p-value*	Median (Range)	p-value*
Gender	Male (n = 39)	59 (0–2128)	0.127	45 (8–1432)	0.705
	Female (n = 27)	55 (16–248)		58.54 (14–58)	
HCC	Present (n = 17)	70 (23–2128)	0.837	45 (18–1432)	0.895
	Absent (n = 49)	61.5 (16–284)		44 (14–401)	
Alcohol consumption	Yes (n = 6)	42.5 (16–73)	**0,039**	25.5 (18–54)	**0.031**
	No (n = 60)	70 (23–2128)		45 (14–1432)	
Autoimmune	Present (n = 9)	90 (35–164)	0.588	52 (32–102)	0.601
	Absent (n = 55)	69.5 (16–2128)		42 (14–1432)	
Cryptogenic	Present (n = 11)	50 (24–284)	0.357	38 (14–401)	0.318
	Absent (n = 57)	70.5 (16–2128)		45 (16–1432)	
Cholangiopathy	Present (n = 8)	108 (54–247)	**0.004**	108 (45–159)	0.103
	Absent (n = 58)	57.5 (16–2128)		39.5 (14–1432)	
HBV infection	Yes (n = 17)	54 (23–2128)	0.459	36 (18–1432)	0.463
	No (n = 49)	88 (16–284)		52.5 (14–401)	
HCV infection	Yes (n = 20)	84 (32–2128)	0.581	46.5 (16–1432)	0.276
	No (n = 46)	58 (16–284)		42 (14–401)	
p-Value**		0.525		0.4	

*Mann-Withney U test.

**Kruskal-Wallis test

### Identification of OBI cases

**Fig 2:**
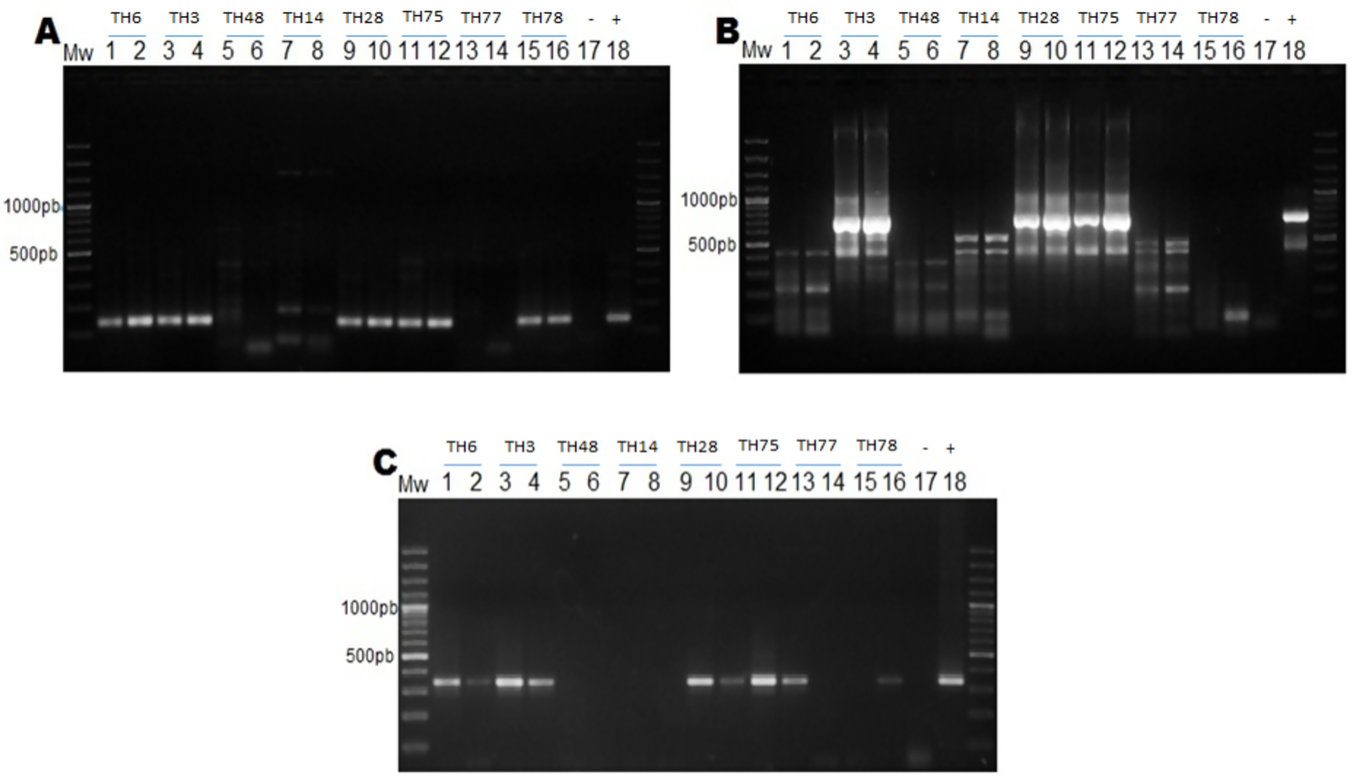
Identification of OBI cases in liver tissue samples from patients with end-stage liver disease. Amplification of HBV X, X-Core and S regions in liver tissue samples obtained from patients without serological evidence of HBV infection. A: ORF X (138 nt); B: ORF Core (729 nt), and C: ORF S (310 nt). All samples were tested at a DNA concentration of 60–80 ng (1-3-5-7-9-11-13-14-15) and by a 1:10 dilution (2-4-6-8-10-12-14-16).

A total of 50 liver samples obtained from patients negative for serological markers HBsAg and/or anti-HBc antibodies were analyzed for HBV genome amplification. The fragments of ORF S and ORF X were successfully amplified in samples TH3, TH6, TH28, TH75, TH78 (Fig 2A and 2C); while ORF Core was amplified in 3 of them (Fig 2B). OBI cases were identified in samples from patients negative for HBsAg and positive for at least two out of the three PCR strategies used for the HBV genome. The HBV genome was also detected by Southern blot in samples TH3, TH6, TH28, TH75 and TH78 (S2 Fig).

Based on the OBI case criteria described by Brechot et al. [52], 5 cases of OBI (5/50, 10%) were identified in the study population. Two OBI cases corresponding to patients with cryptogenic cirrhosis, two patients with cirrhosis associated with HCV infection and one patient with a diagnosis of cirrhosis and HCC associated with HCV infection (Table 3).

**Table 3:** Demographical and clinical characteristics of patients with OBI.

Code	Sex	Age	End-stage liver disease	Etiology of liver disease	Anti-HBc/Anti-HBs
TH3	Male	68	Cirrhosis	HCV infection	Neg/Neg
TH6*	Male	48	Cirrhosis + HCC	HCV infection	ND
TH28	Female	44	Cirrhosis	Cryptogenic	ND
TH75**	Male	34	Cirrhosis	HCV infection	Neg/Neg
TH78	Male	61	Cirrhosis	Cryptogenic	Neg/Neg

ND: not determined.

*Patient undergoing second liver transplant.

** Patient undergoing third liver transplant.

No differences were observed between OBI cases and HBV infection regarding gender, age and ALT and AST serum level (Table 4)

**Table 4:** Demographical and clinical background of patients with OBI and chronic HBV infection.

Variable	HBV (n = 17)	OBI (n = 5)	p-value
Sex (male)	68.75%	80%	0.85^a^
Age mean (SD)	56 (9.3)	51(13.7)	0.718^b^
AST median (IR)	54 (37.5–81.5)	50 (42,5–140) 66 (46.2–130)	0.67^c^
ALT median (IR)	36 (26–65.5)	39 (29.5–124.5) 45.5 (33.7–119)	0.62^c^

ALT: alanine aminotransferase; AST: aspartate aminotransferase. SD: Standard deviation, IR: Interquartile range.

^a^ χ^2^ test.

^b^ t student test.

^c^ Mann-Whitney U test.

### Genotyping OBI cases

Using PCR strategies to amplify the viral genome between 55% and 99% of the full-length viral genome sequences were obtained from OBI samples e.g., TH3: 83%, TH6: 97%, TH28: 70%, TH75: 99%, and TH78: 55% based on the NC009937 reference sequence.

**Fig 3:**
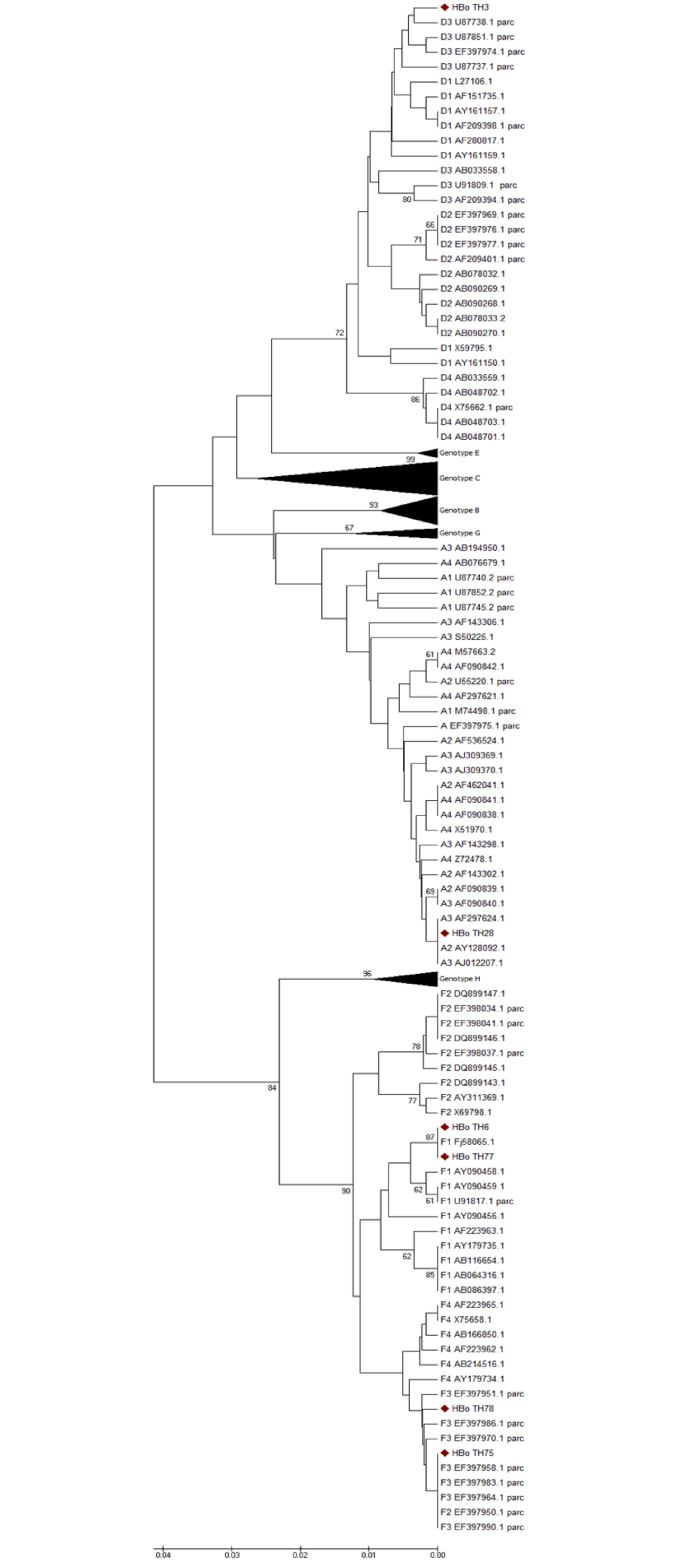
Phylogenetic analysis of OBI sequences. Unrooted phylogenetic tree generated by Neighbor Joining method of partial (part) and full-length HBV sequences. The sequences of this study (TH3, TH6. TH28, TH75, TH78) indicated with black circles, were compared with representative sequences of all HBV genotypes (A-H), denoted with the genotype, GenBank accession number, and country. The genotype of the collapsed blanches is indicated and the bootstrap values from 1000 replicates are indicated in the internal nodes.

The genotyping analysis was performed using partial HBV ORF and complete sequences and evaluated by Maximum likelihood (ML), Neighbor Joining (NJ), and Maximum parsimony (MP). Between the methodologies, the intergenotype branching was conserved and allow us to identify sample TH6 to genotype F, subgenotype F1, while TH75 and TH78 belong to genotype F, subgenotype F3, TH28 to genotype A, subgenotype A2, and TH3 to genotype D, subgenotype D4 (Fig 3).

### Amino acid substitutions in cases of OBI cases

All amino acid (aa) sequences were deduced and compared with reference sequences of HBV genotypes A, D and F. The changes found in each aa sequence are shown in Table 5 (ORF S), Table 6 (ORF P) and Table 7 (ORF X, ORF Core and DNA changes of preC/C promoter).

**Table 5:** HBsAg mutations found in OBI cases.

Sample code	HBV Genotype	HBsAg		
		PreS1	PreS2	S
TH3	D	N37D		**Q51E** **N52K** **C60Y** **G70T** R73H [66] **I255M** **F259V**
TH6	F1	N37D		I195M [67] W196S [68]
TH75	F3	N37D		**C76F** **C99stop**
TH78	F3	N37D		I195M [67]

Positions are based on the reference sequence NC003977. Mutations were identified based on the genotypes A, D and F reference sequences. Newly reported changes are in bold type

**Table 6:** Viral polymerase mutations characterized in OBI cases.

Sample code	Genotype	P protein domains		
		TP	S	RT
TH3	D	R29N	Q219R	**rtP59R** **rtF61I** **rtH91D** **rtL94R**
TH6	F1		Q219R	rtL180M [67] rtM204V [68] rtP237T [69, 70] rtN238S/A [70, 71]
TH28	A			rtL217R [72]
TH75	F3		Q219R	**rtS78T** rtP237T [69] rtN238S/A [70, 71]
TH78	F3		Q219R	rtL180M [67]

Positions are based on the reference sequence NC003977. Mutations were identified based on the genotypes A, D and F reference sequences. Newly reported changes are in bold type

**Table 7:** ORF X, ORF C and Core promoter mutations identified in OBI cases.

Sample code	Genotype	X	HBeAg/Core	Core Promoter
TH 3	D		**V115G** **R127L** **R150I** **R151L** **R154W** **P156R** **R157Q** **R158S** **T160I** **S162Stop** **R166Q** **R167K** **S168P**	
TH28	A	F151L		
TH75	F3	K130M V131I F132Y		A1762T[73] G1764A[73] C1766T[74] T1768A[74]
TH78	F3		**R175G**	

Positions are based on the reference sequence NC003977. Mutations were identified based on the genotypes A, D and F reference sequences. Newly reported changes are in bold type.

Regarding the HBsAg sequence, only one missense mutation, N37D, was found in the preS1 domain in samples TH3, TH6, TH75 and TH78 (Table 5). However, this mutation is located in the receptor-binding domain although; there are no reports of this mutation in the literature.

Moreover, 11 amino acid changes were identified in the S domain of HBsAg, seven in the TH3 (Q51E, N52K, C60Y, G70T, R73H, I255M, F259V), two in the TH6 (I195M, W196S), two in TH75 (C76F, C99stop), and one in the TH78 (I195M) liver sample. Interestingly, no mutations were found in this domain in sample TH28 and only three of them I195M, W196S and R73H were previously reported.

None of the HBsAg mutations described in this study modify the “a” determinant and the major hydrophilic region (MHR), although, the overlapping sequence coding for P protein was altered. A single mutation was recognized in both the TP and S domains of P protein, R29N in the TP domain of the TH3 sample and Q219R in the S domain of TH3, TH6, TH75 and TH78 samples (Table 6). These mutations have not been previously reported and its relation with OBI is unknown. Interestingly the mutation Q129R, corresponding to N37D of the preS1 domain was identified in four OBI cases. This finding raises the question concerning the consequences of the A2915G mutation in OBI pathogenesis and the effects on the P (Q219R) and/or HBsAg (N37D) proteins.

Previously reported mutations targeting the P protein were also identified in this study; these mutations are related to resistance to antiviral therapy, and to increase or decrease of polymerase activity (rtL180M, rtM204V, rtP237T, rtN238S/A, rtL217R) (Table 6). Interestingly, although the OBI patients never received antiviral treatment for HBV and/or HIV infection, Lamivudine and Adefovir resistance mutations (rtL180M, rtM204V, rtL217L, and rtP237T) were detected in 4 OBI samples; these mutations are prevalent in patients undergoing antiviral treatment [69, 73–75].

The presence of mutations in HBx, HBeAg, HBcAg, and the Core promoter is also remarkable (Table 7). Regarding the Core promoter sequence, the four mutations A1762T, G1764A, C1766T and T1768A were found, that had been previously reported [69, 73–75].

### HBV integration events in OBI

**Fig 4:**
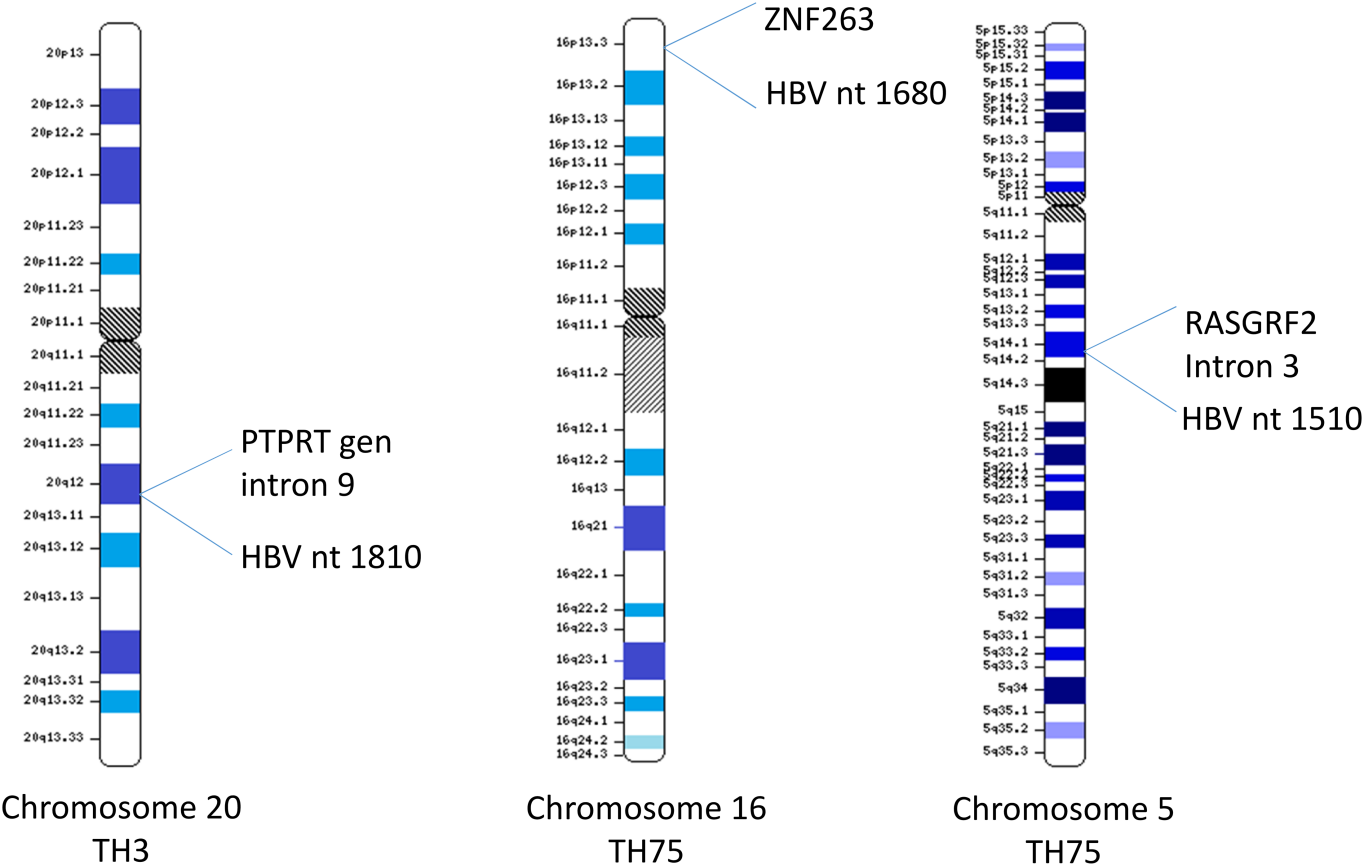
HBV integration events of in cases of OBI cases. Integration sites are indicated with the approximate location in the ideograms of chromosomes 5, 16 and 20 based on G-banding and the sequence of integration (based on NCBI map viewer).

Integration events were identified in samples TH3 and TH75, being characterized three integration events (Fig 4). Among them, one was detected in the TH3 sample mapped in chromosome 20q12, intron 9 of tyrosine phosphatase receptor type T (PTPRT) gene. While two integration events were detected in TH75 sample: one at chromosome 5q14.1, intron 3 of Ras protein-specific guanine nucleotide-releasing factor 2 (RASGRF2) gene and other on chromosome 16p13, in the Zinc Finger 263 (ZNF263) gene sequence (Fig 4).

## Discussion

Colombia is a country with low prevalence for HBV infection considering the average incidence reported (3.11 to 4.68/100.000 habitants); however, in 2015 the incidence range was from 1 to 18.3/100.000 between the states showing a high heterogeneity along the territory. In the state of Antioquia where most patients included in this study are from, the incidence in 2015 was estimated to be 6.6 cases /100.000 habitants [76].

The prevalence of OBI in the Colombian population has been described in some studies carried out on serum samples from HIV patients by Ramirez and Correa (2%, 1/50), by Polo et al. (0.97%, 1/103) and by Bautista et.al (8.7%, 24/275) [29–31]; while in serum samples from Colombian blood donor populations, Beltran et al., Arroyave et al., Castellanos et al., and Rios et al., reported 0% (0/129), 3.4% (7/207), 8.75% (14/160) and 2% (6/302) of OBI, respectively [32–34, 77]. Recently, we described a frequency of 13.3% of OBI cases in 15 liver samples obtained from patients with diagnosis of cirrhosis and/or HCC [36].

In this study we describe the genetic characteristics of OBI cases in Colombian patients with diagnosis of end-stage liver disease. Reports of HBV genome detection have been published since 1990 with a frequency ranging from 4.8% to 72.7% [52]. The first studies performed in Europe, China and Israel, reported OBI in 55.5% [78], 60.7% [79] and 72.7% [80] of liver samples (frozen or paraffin-embedded tissues) obtained from patients negative for HBsAg.

OBI detection studies using liver tissues have performed in populations from China and Brazil. Wong et al. reported 62% of OBI in a cohort of 48 HCC patients among them 80% of cryptogenic cases, 3% of HCV infection and 17% of alcohol related HCC [26]. On the other hand, Ferrari et al. reported 4.4% of OBI in 62 paraffin-embedded liver tissue samples obtained from patients with diagnosis of liver cirrhosis [47]; Similarly, Barros-Junior et al. reported 17.64% of OBI in 17 liver biopsies obtained from patients diagnosed with chronic hepatitis B [81].

The OBI frequency described in the present study population (10%) is similar to other reports of OBI in a country of the region, Brazil (4.4% to 17.64%) that showed the impact of this infection in patients with diagnosis of end-liver disease [28, 48, 49, 82].

One of the most common risk factor of OBI is HIV and/or HCV infection. The frequency of OBI in patients with HCV infection has been reported to be between 33% and 50%, and between 22% and 73.3% in cases of HCV infection and HCC cases related to HCV [49, 83, 84]. The link between HCV and OBI has been clearly established since the HCV core protein inhibits HBV gene expression, by interaction with HBx, and HBV replication by obstructing encapsidation of the pregenomic RNA [85].

In the present study, 60% (3/5) of the OBI cases were identified in patients with HCV infection (two patients diagnosed with liver cirrhosis and one with HCC); the other 40% (2/5) OBI cases were detected in patients with cryptogenic cirrhosis (Table 3). These results are agree with previous reports of OBI with HCV coinfection and cryptogenic liver disease [21, 48, 49, 82, 86].

Based on data presented in Table 1, OBI was identified in 18.2% of cryptogenic cases and in 21.4% of HCV cases. Taking these results in account, OBI diagnosis should be considered in patients with cryptogenic or HCV associated end-stage liver disease before liver transplantation in order to decide what therapeutic strategy should be applied for these patients [47, 48, 87].

The studies of HBV genotyping in different populations revealed that in the Andean region of Colombia, genotype F, subgenotype F3, is the most prevalent, while genotype F1b is the most frequent in Amazonas state (South East) and genotype A is the most common in the Pacific region of the country; even though genotypes D, C, G and E have also been described in the Colombian populations [32, 61, 88–91]. In the present study, the genetic analysis of the viral genome revealed the predominance of genotype F (three cases); subgenotype F1 was identified in one case while F3 was identified in two cases. On the other hand, less common genotypes in the Colombia population, genotypes A and D were also identified in one case each. Currently, there is no evidence of higher OBI risk related with a particular HBV genotype; although variability in CpG islands between genotypes could have an impact on OBI.

Several mutations have been related to OBI; among them, are those affecting the “a” determinant and pre-S domain, that reduce HBsAg immunogenicity and expression, respectively [92–95]. Although other mutations could be related to the outcome of OBI including those affecting the RT domain of P protein (affecting the efficiency of viral replication), inducing the expression of the truncated HBx protein (affecting the normal function of HBx), and altering the promoter activity (downregulation of mRNA expression) [66, 96].

Despite the fact that mutations are well described in OBI cases [66, 85], as in this study (Tables 5–7), the relation between specific mutations and OBI pathogenesis is still controversial [74]. One hypothesis attempting to explain the role of mutations in OBI is the selection of variants as a consequence of host-virus interaction; that is not the case of primary occult infection described in the woodchuck hepatitis virus model that depends on some quasispecies present in the inoculum [97, 98]. Therefore deeper analyses *in vitro* and *in vivo* are required to validate the role of mutations in OBI pathogenesis.

In our study, 25 mutations along the viral genome were detected in the OBI samples. Interestingly, not a single mutation has been identified in all OBI cases or in OBI cases previously described in Colombian blood donors [32]. This finding is in agreement of a number of different mutations described in OBI cases [8, 32, 85, 99], and the mutation rate of HBV (2x10^-5^ nt/generation) [100].

In preS1, N37D, a mutation not previously reported and located in the recognition site for the viral receptor sodium taurocholate cotransporting polypeptide (NTCP) was detected [101]. This mutation may potentially impair the interaction between HBsAg and this receptor by a change of a polar uncharged side chain for a negatively charged chain.

In HBx two mutations were identified, F151L and F132Y, that correspond to changes between two hydrophobic amino acids. The mutations characterized in PreS1 and HBx have not been previously reported, and their role in OBI pathogenesis has to be addressed.

Interestingly, the amino acid sequence of HBc in the TH3 sample shows a combination of mutations in the C-terminus of the protein; this domain is characterized by an arginine-rich region with multiple SPRR motifs, that are important for the interaction with nucleic acids, pgRNA encapsidation and synthesis of the positive strand DNA [102, 103]. As is shown in Table 7, nine mutations were found in sample TH3 (R127L R150I, R151L, R154W, P156R, R157Q, R158S, R166Q, R167K), and one in sample TH78 (R175G) that directly affect the SPRR motif of this region, with potential implications on encapsidation and viral DNA synthesis [104].

Interestingly, the point mutations 1762, 1764, 1766 and 1768 located in the DNA-protein interacting region of the Basal Core promoter region and related with the HBx mutations K130M, V131I and F132Y were detected in sample TH75 (Table 7). These mutations have been reported in both chronic and OBI cases and are known to downregulate the expression of HBeAg and upregulate that of pgRNA by creating an additional HNF1 binding site [73–75, 105]; in addition, these mutations have been related to higher risk of HCC development [106, 107].

However, the characterization of the A1762T, G1764A, C1766T, T1768A mutations in OBI cases could seem contradictory considering the low viral load in this infection; further studies are necessary to demonstrate the role of these mutations in OBI. Even more, the overlapping of Basal Core Promoter and the ORF HBx, could affect the activity of this protein by modifying its nuclear transactivation domain [108–110].

In this study, most of the missense mutations are located between nt 350 and nt 750, comprising the HBsAg S and the RT domain of P protein, that are important regions for escape mutants and antiviral resistance [111]. We identified 10 mutations, 5 of them previously reported and related to drug resistance, replication rescue, downregulation of HBeAg expression and modification of HBsAg antigenicity (Table 6) [67–73, 94].

Ten mutations were characterized on the P protein sequence. The mutation rtL180M, related to adefovir and lamivudine resistance, was found in two cases of OBI (samples TH6 and TH78); while rtM204V related to adefovir, lamivudine, entecavir, and telbivudine resistance [112], was found in one of these two OBI cases, along with rtL180M (sample TH6). Other point mutations previously reported and related to adefovir resistance were rtL217R in TH28, while rtP237T and rtN238S/A were found in the TH6 sample; although none of these cases reported HBV antiviral treatment.

Although P mutations have been described in OBI cases [66], the role of these mutations is still under investigation; moreover, polymerase mutation is usually related to low polymerase activity [113, 114], which could be related to low viral load, one of the hallmarks of OBI.

Considering that the ORF S and ORF P overlap, mutations in one ORF may produce nonsynonymous mutations in the other ORF [115]. Indeed, mutations rtL180M and rtM204L in ORF P produce consecutive mutations I195M and W196S in ORF S; these mutations reduce the recognition of HBsAg by antibodies and therefore their detection by immunoassay [116]; the same effect is also described for R73H, although this change does not correspond to a change in the P protein [66, 117].

Integration of the viral genome is an event of HBV infection, although it plays no role in viral replication. Indeed, integrated viral sequences are detected in tumor tissue of HBV-related HCC and in adjacent non tumor tissue, but the molecular mechanisms of HBV integration is unknown [65, 118, 119]. Integration of viral sequences results in the disruption of one or more ORFs, in deletions, cis/trans activation and expression of truncated protein with novel activity [107, 120, 121].

In this study, three integration events were identified in two liver samples. In sample TH3, the break point of the HBV genome occurs in position nt1682, and was integrated in chromosome 20q12 (Fig 4), intron 9 of PTPRT gene. PTPRT is a member of the protein tyrosine phosphatase (PTP) family. The PTP proteins regulate cell growth, differentiation and mitotic cycle and were identified as potential tumor suppressors genes in colorectal cancer; however, sample TH3 was obtained from a patient without HCC diagnosis. Interestingly, the integration events described in the present study have not been previously reported [122, 123].

In sample TH75, two integrations events were characterized; the HBV genome break point was located in nt 1513 and was integrated in chromosome 5q14, intron 3 of the Ras protein-specific guanine nucleotide-releasing factor 2 (RASGRF2) gene. The second integration event was located in nt 1680 of the HBV genome and was integrated in chromosome 16p13, in the zinc finger 263 (ZNF263) gene (Fig 4).

The RASGRF2 protein is a calcium-regulated nucleotide exchange factor activating RAS and RAS-related protein, that coordinates the signaling of distinct mitogen-activated protein kinase pathways [124]. Interestingly, RASGRF2 expression is usually suppressed in various human cancers [125]. Finally, ZNF263 is a C2H2 zinc-finger protein, containing nine fingers and one KRAB domain [126].

All the integration events occurred in exonic sequence of the genes, potentially impairing the correct mRNA maturation. Beyond the predicted effect of the integration events, functional studies should be made to identify the consequences of these integration events.

Integration of complete or truncated sequences of HBsAg could alter the ratio of ORF S transcripts, affecting the assembly and secretion of virions and subviral particles. Indeed, increased expression of HBsAg L could induce accumulation, and, subsequent reduction of HBsAg secretion [127, 128]. These events may explain the low viral load and lack of detection of HBsAg in occult cases.

## Conclusions

This study is the first characterization study of OBI in patients with end-stage liver disease in Colombia to provide data on HBV genotype, mutations and integration events in OBI cases. We could detect the HBV genomes in 10% of liver samples obtained from transplant recipients. The HBV genotypes F (F3, F1), A (A2) and D (D4) were characterized. In addition, a considerable number of mutations were identified in the OBI cases; among them, the mutation A2915G affecting both the P and HBsAg sequences was identified in 4/5 OBI cases. Interestingly, we also identified antiviral resistance-related mutations and preC/Core promoter mutations; however, none could be associated with low fitness viral replication or impaired expression of HBsAg. In addition, three integration events were characterized in two OBI samples; those events were identified in PTPRT, RASGRF2, and ZNF263 genes.

The limitations of this study are the sample size, the detection limit of PCR strategies for the detection and sequencing of the viral genome and the methodological approach to detect integration only downstream of nt 1130 of the HBV genome; indeed, it has been reported that region nt 1513 to nt 1680 is a common break point for integration [119].

Further studies are needed to determine the prevalence of OBI in different populations in Colombia, as well whether the mutants and integration events described in the present study might participate in the pathogenesis of OBI.

## Supporting information

S1 Fig Complete genome sequencing strategies. Overlapping primers of four PCR strategies were used to amplify the complete genome of HBV.
(DOCX)

S2 Fig HBV DNA detection by southern blot. Total DNA from OBI identified samples was tested for HBV detection using DIG-labeled probe of 319bp targeting S gene was used to detect viral DNA. Line 1: pJET-TH24-1,5, 2: TH3, 3: TH6, 4: TH28, 5: TH78, 6: TH75. Plasmid pJET-TH24-1,5 (10μg) and total DNA (40–50μg) were used.
(DOCX)

S1 Table Primers used for HBV genome detection and sequencing. (DOCX)

S2 Table List of sequences used for phylogenetic analysis. (DOCX)

S3 Table List of sequences used for pairwise mutation analysis. (DOCX)
