# Supplementary figures and images for: Molecular characterization of occult hepatitis B virus infection in patients with end-stage liver disease in Colombia
Source: PLoS One. 2017 Jul 7;12(7):e0180447. doi: 10.1371/journal.pone.0180447 (PMC5501523; doi:10.1371/journal.pone.0180447)

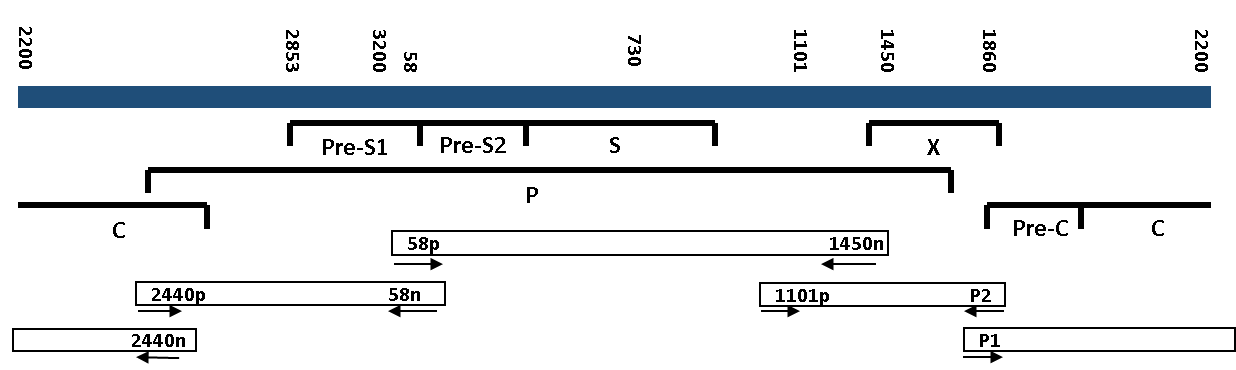

Supplement: S1 Fig — Overlapping primers of four PCR strategies were used to amplify the complete genome of HBV. (DOCX) [file pone.0180447.s001.docx]

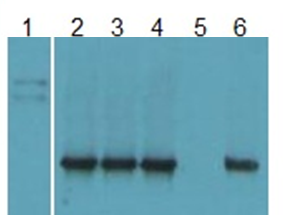

Supplement: S2 Fig — Total DNA from OBI identified samples was tested for HBV detection using DIG-labeled probe of 319bp targeting S gene was used to detect viral DNA. Line 1: pJET-TH24-1,5, 2: TH3, 3: TH6, 4: TH28, 5: TH78, 6: TH75. Plasmid pJET-TH24-1,5 (10μg) and total DNA (40–50μg) were used. (DOCX) [file pone.0180447.s002.docx]
