# Supplementary material for: Molecular characterization of occult hepatitis B virus infection in patients with end-stage liver disease in Colombia
Source: PLoS One. 2017 Jul 7;12(7):e0180447. doi: 10.1371/journal.pone.0180447 (PMC5501523; doi:10.1371/journal.pone.0180447)
Supplement: S1 Table — (DOCX) [file pone.0180447.s003.docx]

**S1 Table. Primers used for HBV genome detection and sequencing**

| **Name** | **Sequence** | **Position** | **Reference** |
| --- | --- | --- | --- |
| YS1 | 5’-GCG GGG TTT TTC TTG TTG A-3' | 202-220 | Zeng GB 2004 |
| YS2 | 5’-GGG ACT CAA GAT GTT GTA CAG-3' | 766-786 |  |
| S3S | 5’-TGC CTC ATC TTC TTR TTG GTT CT-3’ | 421-443 | Schaefer S 2003 |
| S3as | 5’-CCC CAA WAC CAV ATC ATC CAT ATA-3’ | 758-735 |  |
| X3R | 5´- CCCAACTCCTCCCAGTCTTT -3´ | 1733-1721 | Ma NF 2008 |
| X1R | 5´- GGG ACG TCCTTT GTC TAC GT -3´ | 1529-1548 |  |
| X1F | 5´-GGGACGTCCTTTGTCTACGT -3´ | 1410-1429 |  |
| 2440n | 5’-TTG AGA TCT TCT GCG ACG CGG C-3’ | 2430-2409 | Devesa 2008 |
| P2 | 5´-A AAA AGT TGC ATG GTG CTG G-3’ | 1824-1805 | Gunter 1995 |
| 58p | 5’-CCT GCT GGT GGC TCC AGT TC-3’ | 55-74 |  |
| 1450n | 5’-GAT TCA GCG CCG ACG GGA CGT A-3’ | 1446-1425 |  |
| 58n | 5’-GAA CTG GAG CCA CCA GCA GG-3’ | 74-55 |  |
| 2440p | 5’-GCC GCG TCG CAG AAG ATC TC-3’ | 2409-2428 |  |
| 1101P | 5’-CTC GCC AAC TTA CAA GGC CTT TC-3’ | 1097-1119 |  |
| P1 | 5´-T TTT TCA CCT CTG CCT AAT CA-3’ | 1820-1840 |  |
| A5 | 5´ CAGUGCCAAGUGUUUGCUGACGCCAAAGUGCUGGG  AUUACAG 3´ | 1170-1192 | Minami 1995 |
| A3 | 5´ AGUGCCAAGUGUUUGCUGACGACUGCACUCCAGCC  UGGGCGAC 3´ | 1171-1191 |  |
| Tag5 | 5´ CAAGTGTTTGCTGACGCCAAAG 3´ |  |  |
| HB1 | 5´ ACAUGAACCUUUACCCCGUUGC 3´ | 1132-1153 |  |
| HB2 | 5´ GCGCTGCAGTGCCAAGTGTTTGCTGACGC 3´ | 1165-1193 |  |
