# Supplementary material for: Molecular characterization of occult hepatitis B virus infection in patients with end-stage liver disease in Colombia
Source: PLoS One. 2017 Jul 7;12(7):e0180447. doi: 10.1371/journal.pone.0180447 (PMC5501523; doi:10.1371/journal.pone.0180447)
Supplement: S2 Table — (DOCX) [file pone.0180447.s004.docx]

**S2 Table. List of sequences used for phylogenetic analysis**

| Genotype A | Genotype B | Genotype C | Genotype D | Genotype E | Genotype F | Genotype G | Genotype H |
| --- | --- | --- | --- | --- | --- | --- | --- |
| A1 KX264603 Bra | B1 AB073854 Jap | C1 AB014394 Jap | D1 AF151735 Ger | E AB091255 Iv.Coast | F1 AB086397 Jap | G AB056513 USA | H AB059660 USA |
| A1 JQ023660 part Col | B1 D00329 Jap | C1 AF411411 China | D1 AF209398 part Ger | E AB091257 part iv.Coast | F1 AB116654 Jap | G AB056515 USA | H AB064315 USA |
| A1 JQ023662 part Col | B1 D23678 jap | C1 M12906 Jap | D1 AF280817 China | E AB091262 part Iv.Coast | F1 AF223963 Arg | G AB064313 USA | H AF369542 part Mex |
| A1 KF922426 part RSA | B2 AB073827 China | C1 V00867 Jap | D1 Y07587 Ger | E AF323621 part Nig | F1 AY090459 C.Ric | G AF160501 USA | H AY090460 USA |
| A1 KJ533388 part Ind | B2 AB073828 China | C1 X01587 Jap | D2 AB078033 Jap | E FJ692547 Hai | F1 AY179735 Arg | G AF241408 Viet |  |
| A1 AF297621 RSA | B2 AF121246 Viet | C1 X14193 Kor | D2 AB090270 Jap | E L29017 part Sen | F1 EF398033 part Col | G AF405706 Ger |  |
| A1 M57663 Phi | B2 AY217356 China | C1 Y18856 China | D2 AF065119 part Ger | E X75664 Sen | F1 U91817 part C.Ric | G KF414679 Ven |  |
| A1 KM519453 part RSA | B3 AB033554 Indo | C2 AB074755 Thai | D2 AF209401 part Ger |  | F1a FJ589065 Col | G M74499 part Fra |  |
| A1 JQ023660 Col | B3 D00331 Indo | C2 AF068756 Thai | D2 JN688717 Arg |  | F1b JN688691 Arg |  |  |
| A2 AF462041 korea | B4 AB100695 Viet | C2 AF223960 Malay | D2 EF397969 part Col |  | F1b HM585192 Chi |  |  |
| A2 AY128082 Can | B4 M54923 Sula | C2 AY217371 China | D2 X72702 Ger |  | F2 AY311369 Hond |  |  |
| A2 AB937798 part Jap |  | C3 X75656 Poly | D2 Z35716 Pol |  | F2 DQ899145 Ven |  |  |
| A2 HE576988 part Fra |  | C3 003977 | D3 AF061523 part Ger |  | F2 EF398041 part Col |  |  |
| A2 HM467767 Col |  |  | D3 FJ692507 Hai |  | F2 X69798 Bra |  |  |
| A2 X51970 Ger |  |  | D3 KP090180 Bra |  | F2a KX264660 Bra |  |  |
| A2 AF090842 Bel |  |  | D3 U91804 part C.Ric |  | F3 DQ899148 Ven |  |  |
| A2 AF143302 Ger |  |  | D3 U91809 Iv.Coast |  | F3 FJ589066 Col |  |  |
| A2 AJ012207 Ger |  |  | D3 EF397974 part Col |  | F3 EF397951 part Col |  |  |
| A2 KJ854710 part Bra |  |  | D4 AB033559 Papua |  | F4 AB214516 Bol |  |  |
| A2 KP033193 part Bra |  |  | D4 AB048703 Aus |  | F4 KX264662 Par |  |  |
| A2 colvh3 |  |  | D4 AY057948 Tibet |  | F4 X75658 Fra |  |  |
| A2 colvh9 |  |  | D4 FJ692533.2 Hai |  | F4 AB036907 Ven |  |  |
|  |  |  | D4 X75662 part Fra |  | F4 JN688709 Arg |  |  |
|  |  |  |  |  | F3 AP169 |  |  |
|  |  |  |  |  | F3 AP533 |  |  |
|  |  |  |  |  | F3 colvh5 |  |  |

Partial sequences: Part. Arg: Argentina, Aus: Australia, Bel: Belgium Bol: Bolivia, Bra: Brazil, Can: Canada, Col: Colombia, C.Ric: Costa Rica, Chi: Chile, Fra: France, Ger: Germany, Hai: Haiti, Hond: Honduras, Ind: India, Indo: Indonesia, Iv Coast: Ivory Coast, Jap: Japan, Kor: Korea, Mala: Malawi, Mex: Mexico, Nig: Nigeria, Pap: Papua, Par: Paraguay, Phi: Philippines, Pol: Poland, Poly: Polynesia, RAS: Republic of South Africa, Salv: El Salvador, Sen: Senegal, Sula: Sulawesi, Thai: Thailand, USA: United States of America, Ven: Venezuela, Viet: Vietnam
