# Supplementary material for: Molecular characterization of occult hepatitis B virus infection in patients with end-stage liver disease in Colombia
Source: PLoS One. 2017 Jul 7;12(7):e0180447. doi: 10.1371/journal.pone.0180447 (PMC5501523; doi:10.1371/journal.pone.0180447)
Supplement: S3 Table — (DOCX) [file pone.0180447.s005.docx]

**S3 Table. List of sequences used for pairwise mutation analysis.**

| **Accession Number** | **Country/year** | **type of infection** |
| --- | --- | --- |
| AJ012207 | Germany/1998 | NA |
| AJ309369 | France/2001 | Chronic |
| AF090838 | France/1998 | Chronic |
| AF280817 | China/2000 | Chronic |
| AB033558 | Japan/1999 | Acute |
| AB078033 | Japan/2002 | Chronic |
| FJ589065 | Colombia/2008 | Chronic |
| DQ899145 | Venezuela/2006 | Chronic |
| AY179734 | Argentina/2002 | NA |

NA= not available
